# Left Ventricular Assist Device Thrombosis: Combined Approach by Echocardiography and Logfiles Review for Diagnosis and Management

**DOI:** 10.21470/1678-9741-2021-0139

**Published:** 2022

**Authors:** Nicola Vitale, Tommaso Acquaviva, Teresa Paola Quagliara, Nicola Di Bari, Giuseppe Capone, Nicola Marraudino, Aldo Domenico Milano

**Affiliations:** 1 Division of Cardiac Surgery, Department of Emergency and Organ Transplantation, Policlinico Hospital, University of Bari, Bari, Italy.

**Keywords:** Heart-Assist Devices, Thrombosis, Echocardiography, Aortic Valve, Tomography, X-Ray Computed

## Abstract

**Introduction:**

Left ventricular assist devices are an established therapy for end-stage heart failure. Follow-up of these patients showed complications, such as thrombosis. Our objective was to evaluate the contribution of echocardiography — in association with HeartWare HVAD online logfiles reviews and lactate dehydrogenase titration — for diagnosis and treatment of thrombosis.

**Methods:**

Seventeen episodes of thrombosis were diagnosed in 8/20 patients with HVAD. Diagnosis was made by trans-thoracic echocardiographic blood flow velocities, logfiles review of power consumption and pump flows, and titration of lactate dehydrogenase. Data were collected at baseline routine control (Group A), during thrombosis (Group B), after thrombolysis (Group C).

**Results:**

Thrombolysis was successful in all cases; one patient died of cerebral haemorrhage. Echocardiographic maximal blood flow velocity near the inflow cannula was 598±42 cm/sec (Group B), 379.41±21 cm/sec (Group C), and 378.24±28 cm/sec (Group A) (P<0.00001). In eight (47%) cases, thrombi were visualized in the left ventricle by three-dimensional modality. Logfiles recordings of blood flows were 9.52±0.9 L/min (Group B), 4.02±0.4 L/min (Group C), and 4.04±0.4 L/min (Group A) (P<00001). Power consumption was 5.01±0.7 W (Group B), 3.45±0.2 W (Group C), and 3.46±0.2 W (Group A) (P<0.00001). Lactate dehydrogenase was 756±54 IU (Group B), 234±22 IU (Group A), and 257±36 IU (Group C) (P<0.00001).

**Conclusions:**

Echocardiography of increased maximal velocity near the inflow cannula is a sign of HVAD obstruction. Logfile reviews provide a clear picture of HVAD obstruction. Combination of echocardiographic data and review of logfiles detects signs of left ventricular assist devices thrombosis leading to a successful treatment.

**Table:** 

Abbreviations, Acronyms & Symbols	
AML	= Anterior mitral leaflet
Ao	= Aortic valve
CT	= Computerized tomography
INR	= International normalised ratio
LDH	= Lactate dehydrogenase
LPM	= Liter per minute
LVAD	= Left ventricular assist devices
PML	= Posterior mitral leaflet
rT-PA	= Recombinant tissue type plasminogen activator
VAD	= Ventricular assist device

## INTRODUCTION

Left ventricular assist devices (LVAD) are an established therapy for end-stage heart failure, either as a bridge to transplant or a destination therapy.

Several models of LVAD are available for clinical use, one of them being the HeartWare HVAD (HeartWare Inc, Framingham, Massachusetts, United States of America). This device has a wide blade impeller featuring three blood flow paths that provide unloading of the left ventricle during the cardiac cycle. The inflow cannula is of titanium, whereas the outflow cannula has an enhanced flexibility being of polytetrafluoroethylene material.

The follow-up of patients with LVAD has shown complications related to the device, such as thrombosis, driveline infection, and bleeding^[1]^.

Pump thrombosis can cause life-threatening device malfunction and embolic strokes. The causes of thrombus formation can be divided into: 1) mechanical — post-surgical ventricular debris, emboli secondary to clots in the left cardiac chambers or inflow cannula malposition —, 2) inadequate anticoagulation or antiplatelet therapy; 3) haematologic, inflammatory, and immunologic adverse responses activated by blood contact with the prosthetic material^[2,3]^.

Generally, the diagnosis of LVAD thrombosis relies on a combination of clinical, laboratory, and instrumental findings^[2]^. Patients present with shortness of breath and very limited tolerance to activity, also elevated plasma levels of lactate dehydrogenase (LDH) and plasma free haemoglobin due to haemolysis. Imaging of thrombosis include echocardiography and computerized tomography (CT)-scan^[4]^. Echocardiography showed reduced cannula diastolic velocity with preserved systolic velocity, resulting in an increased S/D velocity ratio of the inflow cannula^[5]^.

In addition, in case of thrombosis, the device signals alarm due to abnormal power elevation. Online review of logfiles shows sustained pump power surges and increased blood flows^[6]^.

Thrombolysis is the treatment largely carried out for LVAD thrombosis, although by different protocols of administration^[7]^. Removal of thrombotic obstruction allows return to normal ventricular assist device (VAD) flow and power levels.

The aim of this paper was to evaluate the role of echocardiography and its contribution — in association with online logfiles reviews of pump power, flows, and LDH — for the diagnosis and treatment of thrombosis in patients with HeartWare HVAD.

## METHODS

This is a retrospective review of our experience, spanning from January 2013 to January 2019, when HVAD were implanted. Data were collected from patients’ charts after approval of the Institutional Review Board (56/19).

There were 14 males and six females with a mean age of 66±1.5 years. Indication to LVAD included ischaemic cardiomyopathy in 11 patients and dilated cardiomyopathy in nine patients. All cases received HVAD as destination therapy. The implantation was elective in all cases. Twelve patients were in Interagency Registry for Mechanical Assisted Circulatory Support (or INTERMACS) class 3, seven patients were in class 4, and one patient was in class 5 at the time of implantation^[8]^.

All patients underwent weekly follow-up in the outpatient clinic for the first month after discharge from hospital, monthly follow-up for the next three months, and follow-up every three to four months thereafter, unless otherwise indicated. At each control in our unit, patients underwent a clinical examination and laboratory tests, including LDH, haematocrit, haemoglobin, haptoglobin, coagulation, renal and liver function, and procalcitonin for sepsis. A dedicated technician also collected data on the HVAD device. In addition, patients underwent a transthoracic echocardiogram.

In all patients, the anticoagulation protocol was by warfarin with a target international normalised ratio (INR) of 2-3 and daily 100 mg of aspirin, as suggested by the manufacturer^[9]^. Discharged patients underwent routine controls of INR levels at the anticoagulation clinic of referring hospitals.

Patients with a suspected HVAD thrombosis or complication were readmitted to hospital on an urgent basis. Upon admission, they underwent transthoracic echocardiography and LVAD flow assessment.

### Study Protocol

Our protocol included the retrieval of clinical, laboratory, and echocardiographic data from our database recorded at routine visits during the follow-up. At the same time, the device logfiles were recovered.

### Lactate Dehydrogenase

LDH is the most important laboratory parameter for the diagnosis of LVAD thrombosis, as it is a very sensible marker for haemolysis^[2]^. High levels of LDH are a sign of intra-pump thrombosis, whereas levels are within normal ranges in case of pre or post-pump thrombosis^[10]^. In all cases, LDH titration was carried out at the time of thrombosis, after thrombolysis, and at baseline. In our hospital, the normal plasma levels of LDH range between 87-241 IU.

### Echocardiography

Echocardiographic evaluation was carried out using a Phillips Epic 7 machine with X5-1 and X7-2t probes for the transthoracic and transesophageal approach, respectively. The following transthoracic views were applied: parasternal long and short axis, apical four, three, and two chambers, and subcostal. In case the transthoracic echocardiographic window was poor, a transesophageal echocardiogram was considered. The transesophageal views were mid-esophageal and transgastric, both in two-dimensional and three-dimensional real-time modalities.

In a patient with suspected thrombosis, the four-chamber view with the X-plane modality was applied: the echo beam was directed as much as parallel to the position of the inflow cannula, with the aim to obtain an angle < 20° between the Doppler beam and the cannula. First step was the assessment of the correct position of the inflow cannula in the left ventricle: at least 1 cm away from the interventricular septum and 1 cm below the mitral subvalvular apparatus.

**Fig. 1:**
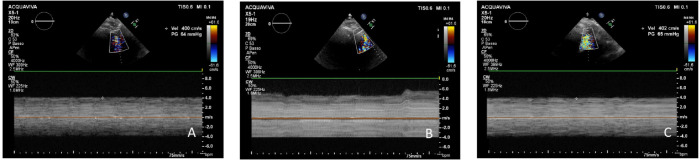
Transthoracic echocardiographic recordings of continuous-wave Doppler flow velocity signals in the same patient. Normal control (A), during left ventricular assist device thrombosis (B), and after thrombolysis (C). Note the intermittent oscillation of flow signal in B due to thrombotic obstruction. No oscillations of the flow signal are present in A and C.

Secondly, the continuous wave colour Doppler function was activated, and the area of aliasing was identified: the continuous wave signal was filtered with the I-scan function, removing the low intensity signals and registering a continuous line. In case of thrombotic obstruction, the continuous Doppler signal showed velocity with an increase of at least 20% than normal velocity, with more frequent and wide oscillations of the line than during the Lavare™ cycle, indicating a partial and intermittent obstruction to the flow through the cannula. These temporary obstructions to blood flow had the secondary effect of increasing its velocity (see Figure 1)^[5,11,12]^. In addition, pulsed

Doppler modality transmitted a very harsh, frequent, and intermittent sound, typical of a thrombosis. The characteristic of this sound was similar to the murmur of aortic stenosis.

Moreover, inflow cannula and left ventricular structures were assessed by three-dimensional modality as well, with the aim to visualize fresh thrombi.

In the presence of a suspected HVAD thrombosis, the ramp test was not carried out because most of the patients presented with dyspnea and might not tolerate low speeds.

### HeartWare Logfiles

HeartWare logfiles are a detailed record of device operation. The controller records three types of logs: data, event, and alarm. Data logs consist of average VAD parameters: rotational impeller speed, power consumption, and estimated VAD flow, which are captured once every 15 minutes. Event logs provide historical information regarding operating parameters that include rotational set speed, blood viscosity, and alarm limit set points. Alarm logs provide historical information regarding activation and clearance of medium and high priority alarm, that includes high Watt, low flow, suction, VAD disconnect, and electrical and controller fault. Both event and alarm entries are recorded at the time of occurrence. In particular, the alarm starts signalling when power consumption is 1.5 Watt above the normal mean Watt consumption, as recommended by the manufacturer.

In case of thrombosis, the site of obstruction of the HVAD may be classified by logfiles readings as pre-, intra-, or post-pump according to the position of blood clots[10]. In the pre-pump thrombosis, thrombi are located at the orifice or within the inflow cannula: logfiles show a drop of flows with normal power consumption. The intra-pump thrombosis is generated by clots lining the impeller: logfiles features are a rapid increase of blood flows and power consumption. The post-pump thrombosis causes the obstruction of the outflow cannula. In this case, a slow decrease of flows and power consumption characterize the logfiles^[10]^.

In our patients, analysis of logfiles was carried out at the time of thrombosis and compared with baseline and after thrombolysis data.

### Thrombolysis

In all cases, thrombolysis was carried out by administration of 100 mg of recombinant tissue type plasminogen activator (rT-PA) according to the following protocol: 10 mg bolus infusion followed by 40 mg over one hour and the remaining 50 mg over two hours. This is the same institutional protocol applied for primary prosthetic valve thrombosis^[13]^. All rT-PA treatments were performed via a peripherally inserted central venous line in the cardiac intensive care unit. Patients were closely monitored for signs of bleeding.

### Statistical Analysis

Data are presented as the mean ± standard deviation for continuous data or as percentages for categorical data. LDH, echocardiographic measurements, and logfiles data were divided into three groups, according to the time of observation. Group A comprises data at baseline, Group B data of patients with thrombosis before thrombolysis, and Group C data of patients after thrombolysis. In the eight patients, the baseline time frame was considered the last echocardiographic and logfiles control exhibiting normal values before the occurrence of thrombosis; in all cases, the routine control before the occurrence of the thrombotic event was reported as normal. Data after thrombolysis were recorded within three hours after the end of rT-PA infusion. Comparisons between data groups were performed using the Student’s *t*-test or Wilcoxon rank sum test for normally and non-normally distributed data for continuous variables, respectively. Fisher’s exact test was applied for categorical variables. Changes in echocardiographic and pump function variables were compared using the Wilcoxon signed rank or paired Student’s *t*-test as indicated. The Bonferroni correction was applied for the adjustment of multiple testing. A value of *P*<0.05 was considered statistically significant.

## RESULTS

A total of 17 thromboses were observed in eight patients, seven males and one female, with a mean age of 65±2.1 years; one patient experienced one event, four patients had two events, one patient had three events, and the remaining patient experienced four events.

Patients were hospitalized within 2-3 hours of suspected VAD thrombosis based on echocardiographic findings or logfiles data collected soon after admission. In eight cases out of 17 (47%), the patients were completely asymptomatic; while in nine cases out of 17 (53%), patients presented with dyspnea and complained of a reduced tolerance to daily activity the previous couple of days. All patients were haemodynamically stable on admission to our unit and did not require infusion of inotropic drugs.

Anticoagulation with warfarin was inadequate in 14 cases, with a mean INR of 1.7±0.3, because patients were not compliant with prescription of the anticoagulation centre. In the remaining three cases, INRs were within therapeutic ranges, but they had stopped the daily intake of aspirin without medical advice.

The patient who experienced four thrombotic events underwent successful heart transplant two months after the last event. In all patients, search for raised plasma procoagulant factors was negative. Two patients had a driveline infection, but no raised laboratory parameters of sepsis were found.

### Thrombolysis

Thrombolysis was successful in all cases. Of note, a rapid, progressive, and constant decrease of power consumption and flows back down to normal values was observed on the HVAD screen soon after the start and during rT-PA infusion.

One patient died of cerebral haemorrhage at the end of treatment. No other bleeding events or complications were reported.

### Lactate Dehydrogenase

Levels of LDH at the time of thrombosis were significantly raised, as a sign of intra-pump thrombosis: 756±54 IU in Group B, compared to baseline and after thrombolysis time frames, 234±22 IU and 257±36 IU in Groups A and C, respectively. A significant difference was found in Group C *vs.* Groups A and B (*P*<0.00001). No difference was found between Groups A and C (*P*=0.5).

### Echocardiography

In all cases, the transthoracic echocardiogram provided satisfactory echo windows, therefore no transesophageal echocardiogram was necessary. In all patients, the orientation of the inflow cannula within the left ventricle and of the outflow cannula was correct. There were no signs of post-pump thrombosis.

**Fig. 2:**
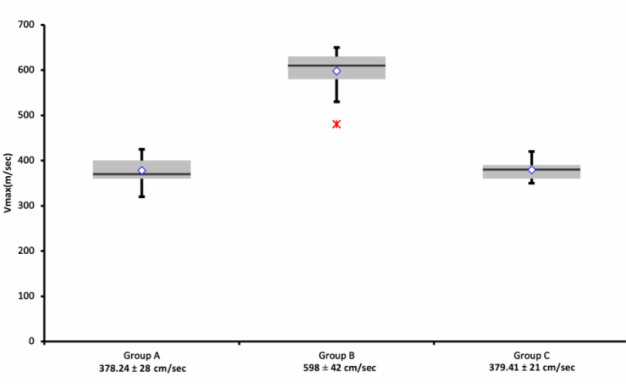
Maximal flow velocity (cm/sec) recorded by transthoracic echocardiography four-chamber view. Data are presented as scatter plots; mean values with standard deviations are at the bottom. Differences in Group B vs. Groups C and A are highly significant (P<0.00001); no significant difference between Groups A and C (P=0.5).

With regard to maximal blood flow velocity in the left ventricle at the near site of the inflow cannula, it was 598±42 cm/sec during thrombosis (Group B), whereas it was 379±21 cm/sec after thrombolysis (Group C) and 378±28 cm/sec at baseline control (Group A). The maximal velocities recorded at the time of thrombosis were as higher as to reach statistical significance compared to baseline and after thrombolysis recordings: Group B *vs.* Groups C and A (*P*<0.00001). On the other hand, no statistically significant different maximal blood velocities were noted in Groups A and C (*P*=0.5). Data and their comparisons are presented as scatter plots in Figure 2.

**Fig. 3:**
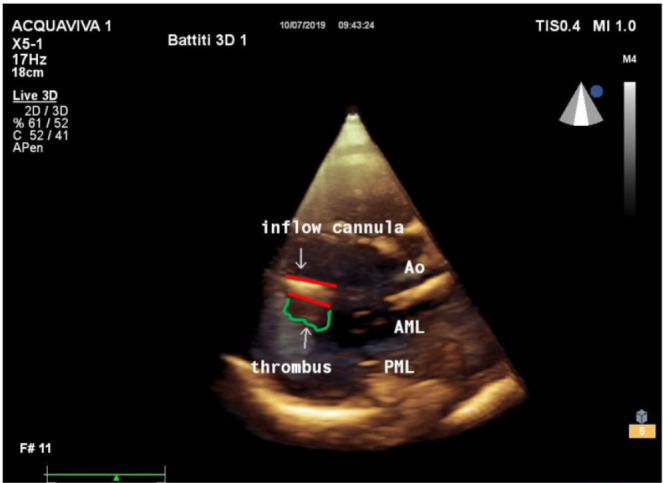
Three-dimensional transthoracic echocardiography. Apical view of the left ventricle: red lines mark both sides of the inflow cannula. The thrombus attached at the bottom side of the cannula is marked in green: the thrombus is not wedging into the cannula, excluding a pre-pump thrombosis. AML=anterior mitral leaflet; Ao=aortic valve; PML=posterior mitral leaflet

At the time of thrombosis, in eight cases out of 17 (47%), thrombi were detected in the left ventricle lining the outer surface of the inflow cannula without wedging into its lumen. No clots were detected after thrombolytic treatment. Figure 3 shows a three-dimensional apical transthoracic view with a thrombus attached to the bottom side of the inflow cannula.

### Logfiles Review

**Fig. 4:**
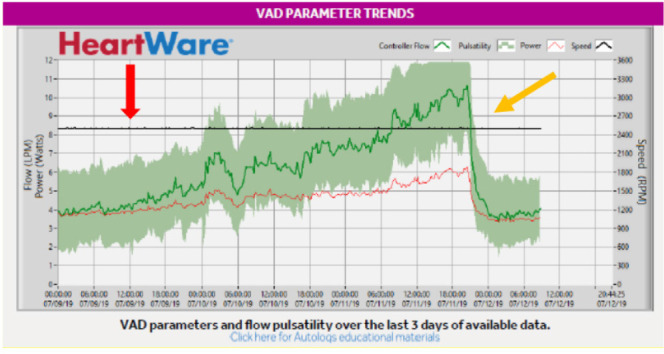
Logfile recording of a thrombotic event in a HVAD. Power consumption (red line) and blood flow (green line) surge abruptly well above normal range (red arrow). These logfiles features are typical of an intra-pump thrombosis. From the beginning of thrombolysis, a decrease of these parameters back down to normal values takes place (yellow arrow). LPM=liter per minute; VAD=ventricular assist device

Alarm signals of power consumption were present in all cases. Flows and power exhibited very rapid and high rise to abnormal values at the time of thrombosis, suggesting an intra-pump thrombosis^[10]^. A marked decrease of both parameters down to normal control levels occurred after thrombolysis (see Figure 4).

In details, online recordings registered the following blood flows: 9.52±0.9 L/min in Group B *vs.* 4.04±0.4 L/min in Group A and 4.02±0.4 in Group C (*P*<0.00001). No significant difference was found between Groups A and C (*P*=0.5).

**Fig. 5:**
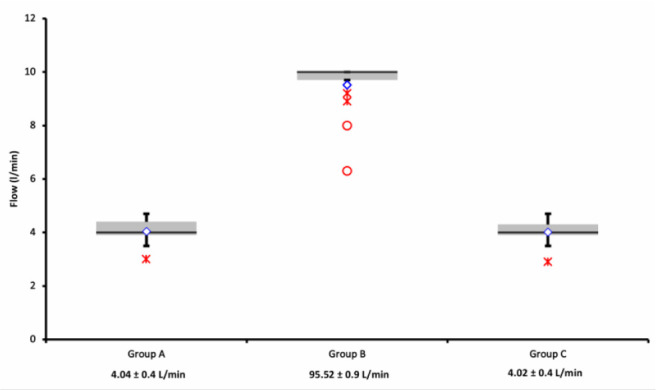
Logfiles review of blood flow (L/min) in the HVAD. Data are presented as scatter plots; mean values with standard deviations are at the bottom. Flows in Group B vs. Group C and Group A (P<0.00001) were significantly higher. No significant difference was found between Groups A and C (P=0.5).

**Fig. 6:**
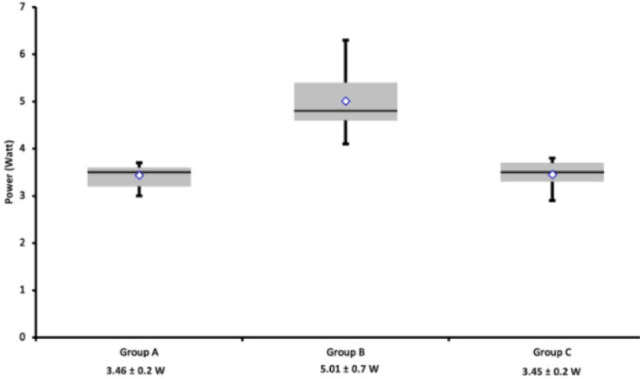
Logfiles review of power consumption (Watt) in the HVAD. Data are presented as scatter plots: mean values with standard deviations are at the bottom. Energy was significantly higher in Group B vs. Groups C and A (P<0.00001); the difference between Group A and Group C did not reach statistical significance (P=0.5).

Power consumption was 5.01±07 W in Group B *vs.* 3.45±0.2 W in Group C and 3.46±0.2 W in Group A (*P*<0.00001). Similar power consumptions were noted in Groups A and C (*P*=0.5). Flow and power data, depicted as scatter plots, and their comparisons are presented in Figures 5 and 6, respectively.

## DISCUSSION

Data from the Randomized Evaluation of Mechanical Assistance for the Treatment of Congestive Heart Failure (or REMATCH) trial indicate that LVAD implantation increases survival and quality of life as compared to optimal medical treatment alone^[14]^. The survival of patients with LVAD continues to improve, but they remain at high risk for fatal complications like pump thrombosis. The incidence of pump thrombosis reported in the initial and extended clinical trials of LVAD ranged from 0.014 to 0.03 events per patient-year, but increased incidences of pump thrombosis have been noted^[1,5,7]^.

The rate of thrombosis in our series was higher than in other series^[1,5,7]^. Najar et al. reported a distinct time related increase in the rate of occurrence of HVAD thrombosis: from 2.2% after the first year to 8.7% after three years from the implant^[1,5,7]^. Similar rates of thrombosis were reported in other series with HVAD and other types of LVADs^[3]^. The reason our rate of thrombosis was higher was due to the unsatisfactory compliance of some patients and that anticoagulation management in between our routine controls was left to peripheral referring hospitals, which were not familiar with this kind of patients. Moreover, two patients had an *in-situ* driveline infection and, despite the negative laboratory results for sepsis, it cannot be excluded that this had a negative effect on anticoagulation.

In our series, the diagnosis and management of pump thrombosis in HVAD patients was based only on LDH levels and echocardiographic and logfiles findings. Unfortunately, quite often, the diagnosis does not rely on the simultaneous evaluation of these three elements. Our simple approach introduced this novelty. It proved successful and led to the rapid, correct treatment and management. The association of raised LDH, echocardiographic evidence of increased maximal flow velocity in the left ventricle in the proximity of the inflow cannula, together with the marked, abnormal surge of power consumption and flow of the device led to the identification of the type of malfunction. This was of the intra-pump type in all cases^[10]^.

Diagnosis of pump thrombosis relies on clinical, laboratory, and instrumental tests. On clinical grounds, patients may present with signs and symptoms of left heart failure, complaining fatigue up to pulmonary oedema. Acute renal failure may be present as well. Laboratory tests include the test for haemolysis, such as LDH, haematocrit, haemoglobin, and haptoglobin. LDH is the most specific indicator of pump thrombosis: elevation > 2.5 should require further investigation^[2]^. In our series, LDH plasma levels had almost > 3 fold increase at the time of thrombosis, indicating an intra-pump thrombosis^[10]^. In case LDH levels do not drop after thrombolysis, LVAD exchange should be considered. Pump thrombosis may determine a decrease of haptoglobin, haematocrit, and haemoglobin too, but these may also decrease due to other causes unrelated to thrombosis (*i.e.*, patient hyperhydration, concomitant presence of a prosthetic heart valve, bleeding)^[2]^. Several authors advocate monitoring of haemolysis for the prevention and early treatment of pump thrombosis during the follow-up. This is based on the observation that pump thrombosis may have a slow onset, and patients develop haemolysis as the first sign^[8]^.

With regard to echocardiographic findings, our series support once more the pivotal role of echocardiography for the detection and management of HVAD thrombosis, as demonstrated by several authors previously^[1,5,11,12,15]^. The assessment of the correct position of the inflow and outflow cannula was achieved. Despite quantification of blood flow velocity at the site of the inflow cannula within the left ventricle is reported difficult to record due to artefacts around the cannula^[11]^, the alignment of the echo probe to the inflow cannula describing an angle < 20° allowed assessment of increased flow velocities. These are an indirect sign of pump obstruction. Moreover, the addition of transthoracic three-dimensional modality identified, in eight cases out of 17 (47%), thrombi lining the outer surface of the inflow cannula without edging into the lumen. Detection of thrombi shed a light on the likely mechanism of HVAD thrombosis: formation of clots occurs in the left ventricle in areas close to the inflow cannula, flow turbulence triggers the fragmentation of clots into debris that easily migrate into the impeller of the device through the inflow cannula, and, as a result, the device increases the energy consumption.

Finally, the use of echocardiography avoids further investigations like the CT-scan because the information acquired by the CT, like position of the inflow and outflow cannula, can very easily be gathered by echocardiography, reducing the time to diagnosis and avoiding administration of contrast medium^[5]^.

As far as the logfiles are concerned, these are a very precious tool in the hands of the physician to understand the underlining problem. Logfiles signs of intra-pump obstruction have very clear features: the sudden and marked abnormal surge of flow and energy consumption and their return to normal levels after treatment are very easy to interpret. Flow may also have an oscillating pattern that may not guarantee a constant, adequate cardiac output, and some patients may present with exertional dyspnea. On the other hand, sudden decrease of flow with steady power consumption are typical features of inflow cannula obstruction, whereas a slow decrease of flow and power consumption characterize outflow cannula obstruction^[10]^. The prompt availability of these recordings is another element that justifies their use. In our series, all cases presented with the same patterns of obstruction on logfiles recordings.

Finally, in our series, LDH plasma levels were > 3-fold increased at the time of thrombosis, in line with results in other series^[2]^. A LDH level > 5 times the normal limit is highly specific (92.5%) and sensitive (100%) for the diagnosis of VAD thrombosis^[2]^. In the series by Birati et al.^[16]^, two patients with confirmed pump thrombosis had a < 3-fold increase of LDH, reducing the sensitivity of 5-fold LDH cutoff to 82%. The likely explanation for the abrupt rise of LDH is that there is turbulent flow and increased shear stress, causing the rupture of red cells and raised LDH serum levels. With regard to LDH isoenzymes at the time of VAD thrombosis, Gordon et al. reported raised LDH-1 and LDH-2 and decreased LDH-4 and LDH-5^[17]^.

In the setting of suspected thrombosis, surgical device exchange or urgent heart transplantation represent the most definitive treatment modalities^[10]^. However, cardiothoracic surgery is not without risks. An additional surgery for pump exchange can result in formation of scar tissue and adhesions, which can increase the duration and risk of bleeding during subsequent surgery for heart transplantation^[3]^. Therefore, it is important to explore medical management strategies to treat pump thrombosis for transplant candidates or patients who cannot withstand surgery.

Thrombolysis has emerged as the medical treatment largely in use^[1,7]^. Unfortunately, there are limited series of patients, and the type of treatment varies greatly, from rT-PA, urokinase to prolonged heparin infusion^[1,7]^.

In our cases, the institutional protocol for primary prosthetic valve thrombosis was adopted^[12]^. Results were satisfactory, with a 100% rate of success. Only one patient died of cerebral haemorrhage at the end of treatment, with the rate of bleeding of this series being 5.8% (1/17), well within or even below the rates of bleeding reported in another series^[18]^. Beside differences about the thrombolytic drugs, there are also discrepancies about the doses of rT-PA administered among the different series^[1,7]^. Several authors reported successful thrombolysis with a minor dose^[1,7]^: it must be said that, in the present series, the drop of flow and consumption energy of HVAD begun soon after the start of the rT-PA infusion, indicating that the first 40-50 mg infused were likely a sufficient dose to dissolve the clots. On the other hand, it is virtually impossible to document that a total lysis of thrombi within the pump impeller has taken place. This is because, apart the echo imaging of clots in the left ventricle, all other measures available for diagnosis of device thrombosis are indirect. Therefore, the infusion of a higher dose of rT-PA may be justified by the effort to guarantee a total clean-up of the impeller by residual thrombi. Nonetheless, further clinical studies monitoring reduction of LVAD power during thrombolysis may provide useful information on the appropriate dose.

The patient in our series who experienced four thrombotic events was successfully transplanted after the last episode. This decision was taken because the patient continued to experience thrombosis, despite the anticoagulation management, and overall follow-up was monitored very closely since the first event. Therefore, it was felt that further events with consequent rT-PA treatments would have raised the risk of mortality and morbidity to such an extent as to consider transplant a safer option.

### Limitations

The limitation of this study is the small population, being not a cooperative trial.

## CONCLUSION

In conclusion, in our series, the simultaneous evaluation by echocardiography, logfiles review, and LDH titration proved new, easy, and reliable for the diagnosis and management of HVAD thrombosis.

**Table:** 

Authors’ Roles & Responsibilities	
NV	Substantial contributions to the conception and design of the work; drafting the work; final approval of the version to be published
TA	Substantial contributions to the conception and design of the work; drafting the work; final approval of the version to be published
TPQ	Substantial contributions to the acquisition and analysis of data for the work; final approval of the version to be published
NDB	Substantial contributions to the acquisition and analysis of data for the work; final approval of the version to be published
GC	Substantial contributions to the acquisition and analysis of data for the work; final approval of the version to be published
NM	Revising the work critically; final approval of the version to be published
ADM	Final approval of the version to be published
